# The actuality and influences of undergraduates’ participation in high impact educational practices in Chinese universities

**DOI:** 10.3389/fpsyg.2022.890493

**Published:** 2022-08-12

**Authors:** Hu Ke, Ni Jianchao, Li Xiaojing, Zhou Xiting

**Affiliations:** ^1^School of Education, Fujian Normal University, Fuzhou, China; ^2^Institute of Education, Tsinghua University, Beijing, China; ^3^School of Aerospace Engineering, Xiamen University, Xiamen, China; ^4^School of Marxism, Fujian Normal University, Fuzhou, China

**Keywords:** “Double First-Class” university, High Impact Educational Practices, learning gains, student engagement, undergraduates

## Abstract

High Impact Educational Practices (HIPs) are effectively educational practices that have an important impact on student learning and development. The purpose of this study is to investigate the impact of different types of high-impact educational activities on students’ learning outcomes. The data comes from the 2019 “tracking research survey on learning and development of Chinese college students,” in which undergraduates from 39 Chinese colleges and universities participated. This study first clarified the concept and classification of high-impact educational activities, and then used multiple linear regression analysis to analyze the impact of three types of high-impact educational activities, including extended learning activities, research-related activities, and social practice activities, on students’ learning gains. It’s found that most Chinese college students do not perform well on HIPs, while the “Double First-Class” university students engage more than other colleges. Participating in HIPs has a significant impact on students’ knowledge, ability and values, especially on the latter two. This study provides valuable enlightenment for universities on how to promote students’ participation in high impact educational activities and improve the quality of higher education.

## Introduction

The total number of students in China’s higher education had reached 44.30 million by 2021, with a gross enrollment rate of 57.8%. Quantitatively, China’s higher education has entered the stage of popularization. At this stage, it is necessary to improve the quality of higher education and realize connotative development to build China into a country high-quality education. Talent cultivation is the core task of the quality construction of higher education, and undergraduate education is the foundation of talent cultivation in colleges and universities. Therefore, comprehensively improving the ability of talent cultivation of colleges and universities is an effective way to speed up the construction of high-level undergraduate education.

In order to improve the quality of talent cultivation, developed countries have launched a “student-centered” education reform movement, focusing on the process and learning results of college students, advocating that colleges and universities should take necessary actions to promote students’ learning. The learning process of students consists of in-class learning activities and extra-curricular activities. The significance of classroom teaching on students’ development is self-evident, but many studies also show that extra-curricular learning activities also have important impacts on students’ development (Seow and Pan, 2014). Especially in today’s diversified learning forms and personalized learning needs. High Impact Educational Practices (HIPs) have become an indispensable education resource for universities, as well as an important part of promoting college students’ learning and improving the quality of education. To this end, the Association of American Colleges and Universities (AACU) identified 10 HIPs that could benefit undergraduates broadly, and it has been incorporated into the Valid Assessment of Learning in Undergraduate Education (VALUE), which has a wide impact in the field of higher education. Kuh and Schneider (2008) carried out investigations and studies on Undergraduates’ participation in HIPs, Since then, A variety of assessment options utilizing high-impact educational practices have emerged to assist faculty in higher education with college student learning outcomes (White, 2018). Under the background of “double first-class” construction, some scholars have begun to explore HIPs for Chinese undergraduates, but the existing research is insufficient, and there is a lack of research on the impact of HIPs on students’ learning gains. Therefore, this study will further explore the current situation of undergraduate participation in HIPs and its impact on students’ learning gains, which is of great practical significance to promote college students’ learning and improve the quality of undergraduate talent cultivation.

## Literature review

### Characteristics and classification of high impact educational practices

There is no strict and clear definition of HIPs. Broadly speaking, educational practices which have a profound impact on students’ learning and promote students’ development can be called HIPs. Although there is no unified definition and standard metric, there are some typical characteristics of these activities. Kuh and Schneider (2008) pointed out that HIPs require students to devote a lot of time and effort to learning tasks, to form more interaction between teachers and students, to interact with students from different backgrounds, to apply their own knowledge to real life and change life. Researchers from China concluded that the HIPs for undergraduates in Chinese universities have the following characteristics: encourages students to carry out extensive and in-depth exchanges with each other; provides students with free thinking space and promotes students to learn independently; designs challenging tasks and provides students with scenarios for cooperation and creativity; requires students to put theory into action and learn in practice; pay attention to the exemplary role of tutors, which can inspire students to think deeply; provide a lot of opportunities for reading or writing; let students experience multiculturalism, etc. (Xu et al., 2020). It can be seen that the value of HIPs is that they can provide students with the opportunity to examine the knowledge they have learned, and create meaningful learning experience for students, thus promoting students’ learning and development.

However, there are a wide range of educational practices with the characteristics of HIPs, and academic circles have also tried to classify them. Kuh and Schneider (2008) classified the activities that students choose independently after class and could effectively promote students’ learning into the top HIPs, including freshman seminar and freshman experience, general experience project, learning community, intensive writing courses, collaborative homework or project, undergraduate scientific research experience, diverse or global learning, service learning or community learning internships, captive-level courses or projects. It was applied in the National Survey of Student Engagement (NSSE), after that, NSSE reduced it to 6 categories: service learning, learning community, research with faculty, internship or field experience, study abroad and culminating senior experience (National Survey of Student Engagement, 2014). Other researchers integrated HIPs into five aspects: namely freshman seminar, learning community, undergraduate scientific research project, service learning project, and vertex learning experience (Brownell and Lynn, 2010). Chinese scholars summarized 6 HIPs, including internship, research with teachers, and overseas study etc. based on the actual situation of the original “985 Project” universities in China (Wen et al., 2014). Some researchers from China constructed a classification system of HIPs with Chinese characteristics based the questionnaire of the China College Student Survey (CCSS) in 2015, and HIPS is integrated into nine activities in three categories, including “extended learning activities,” “research-related activities,” and “social practice activities”(Zhang et al., 2017).

### Research on the influences of high impact educational practices on students’ learning gains

The Joint Committee on Standards for Educational Evaluation defines “student learning outcomes” as “knowledge and understanding (cognition), practical skills (skills), attitudes and values (emotion), and individual behavior that students should acquire after completing courses and degrees” (Arlen, 2003). Kuh and Hu (2001) points out that learning gains refers to the ability of students who can prove that they are equipped with due abilities in knowledge, skills and values after completing a series of courses or training plans, which is the standard to measure students’ development. Learning gains is often mixed with words such as “education gains” and “learning development.” There is no unified concept, but its direction is relatively clear. It usually refers to that students’ learning experience in school, promoting their growth in knowledge, skills and values.

Existing studies have shown that HIPs have an important impact on students’ learning gains to varying degrees. Some researchers analyzed the impact of ten HIPs on the results of general education by using student survey data (divided into pre-test and post-test), the results of this study showed that the HIPs advocated by AACU were an important way for students to achieve student success (Kilgo et al., 2015). Students who have participated in HIPs have shown gains in retention, in persistence, intellectually and in an overall positive college experience (Peters et al., 2019), HIP participation is a significant predictor of future career plans and early job attainment (Miller et al., 2018). Some studies also showed that students’ participation in HIPs might affect students’ ability development. For example, Some researchers found that students’ participation in HIPs (such as sociocultural dialogue with peers, community service, etc.) would affect students’ leadership development (Priest and Clegorne, 2015). Other researchers also showed that students’ participation in service learning was positively correlated with students’ learning achievements, and could improve students’ multicultural ability as well as increasing students’ commitment to social work responsibility (Jones and Abes, 2004). Kuh (2009) believed that the time and effort invested by students are the key to learning gains, and the reason HIPs can promote student success is that these activities emphasize students’ high-intensity learning engagement. At the same time, these activities provided opportunities for substantive communication between teachers and students, cross-cultural learning experience, and the application of theoretical knowledge into practice. Since NSSE was introduced into China, Chinese scholars began to pay attention to HIPs for undergraduates. Ye (2012) introduced American HIPs earlier, believing that it was an effective means for American universities to promote students’ success. Others pointed out that HIPs could stimulate students’ internal motivation in learning, and enhanced students’ learning purpose and initiative, thus improving students’ learning gains (Wen et al., 2014). Other studies have analyzed the characteristics of a generation of college students’ participating in HIPs (Zhang et al., 2017; Liu, 2020), and the matching degree between HIPs and the graduation expectations (Guo, 2019). Although some studies have also found that high-impact practices are in widespread use across different institutional types but have limited relationships with graduation rates (Sarah and Frances, 2018).

In general, research abroad on HIPs for undergraduates are relatively abundant, and many studies also reveal that HIPs have a positive impact on students’ learning achievements such as ability and values. Chinese scholars also began to pay attention to the important role of HIPs in promoting students’ development, and studied the characteristics and influencing factors of HIPs. However, most of the existing studies focus on research universities or special groups (such as a generation of college students), and in-depth studies are still needed on the differences between different types of institutions and different student groups, especially on how HIPs affect students’ learning gains. Therefore, this study will use large-scale data to analyze the impact mechanism of HIPs on students’ learning gains, so as to provide reference for colleges and universities to carry out undergraduate teaching reform and improve the quality of talent cultivation.

### Study on the influencing factors of college students’ learning gains

It should be noted that the learning gains of college students is also affected by individual and environmental factors. To discuss the influence of HIPs on students’ learning gains, individual and institutional factors should be taken into consideration.

#### The influence of individual factors on students’ learning gains

The influence of individual factors on students’ learning gains could be divided into two aspects. Firstly, the influence of students’ ascriptive factors such as gender and family background on learning gains. There is a study that discussed the significant impact of gender and family background on students’ academic performance, especially in the field of STEM, gender differences are widespread (Kabayashi et al., 2020). Weiser and Riggio (2010) found that family background and self-efficacy might affect both Students’ learning gains, while family background might affect students’ self-efficacy. Another study found that students’ learning motivation and learning behavior habits also had a significant impact on learning gains in addition to the inherent external factors (Sun et al., 2012).

#### The influence of college factors on students’ learning gains

In the “input-environment-output” model proposed by Astin (1990), it is believed that in addition to students’ background, the internal environment of colleges and universities would also affect education results. Pascarella (1985) emphasized comprehensive influencing factors, including students’ background and school organizational characteristics, jointly affect students’ learning and cognitive development through students’ personal efforts and school environment. Mahan (2010) found that there was a significant correlation between campus relations, various school environments and learning outcomes. Museus and Chang (2021) took first-generation college students as the research object and studied the impact of the campus environment on the learning gains of these students. It was found that the campus environment had a significant impact on students’ sense of belonging, which in turn affected their learning gains. Other studies have found that there are significant differences in learning outcomes of students in different types of colleges, disciplines and grade. For example, A study found that the higher the level of the institution, the higher the learning gains, and the process factors of students’ learning input had a far greater impact on students’ learning gains than students’ ascribed factors (Zhao et al., 2012).

College Students’ participation in HIPs is often regarded as a part of students’ learning input. Students’ learning input is the main reason affecting learning gains. A study showed that students’ learning input was not only an important factor affecting learning gains, but also an important dimension to evaluate learning quality (Li and Qin, 2019). However, students’ learning input focused on two levels: individuals and institutions. Hu and Kuh (2003) pointed out that the former referred to the time and energy students devoted to learning and other purposeful educational practices, while the latter focused on the teaching resources invested by institutions and the learning support and opportunities provided for students to participate in learning activities. Therefore, in order to explore the impact of HIPs on students’ learning gains, it’s necessary to pay attention to individual and institutional background factors.

### Conceptual model

**FIGURE 1:**
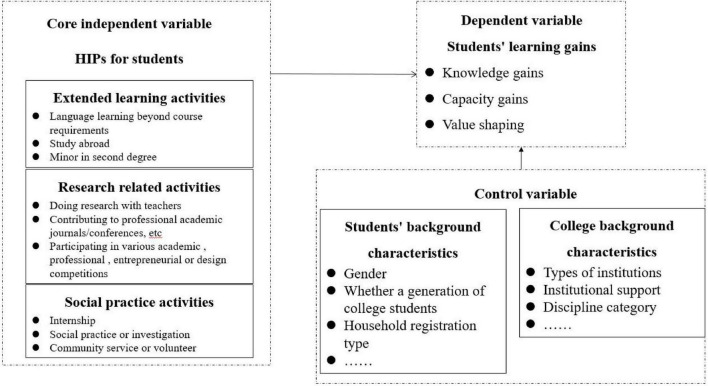
Conceptual model.

In summary, it is urgent to further the study of HIPs for undergraduates, especially the impact of HIPs on students’ gains. Based on the literature research, this study puts forward the following conceptual model (as shown in Figure 1). In view of the complexity of the classification of HIPs, it is not appropriate to be too general or detailed. Therefore, this study will continue to follow the classification of “3 categories and 9 items” of HIPs by Zhang et al. (2017), a member of CCSS research group for the that the concept of “HIPs” comes from the study investment survey of American college students. The setting of HIPs in CCSS questionnaire is consistent with the NSSE survey of the United States. The classification of “3 categories and 9 items” of HIPs not only maintains the essential characteristics of the original concept, but also incorporates the situational elements of Chinese colleges and universities, which has also been verified by large-scale data. This study also summarizes the extended learning activities as learning activities outside the curriculum or major, including language learning outside the curriculum requirements, overseas learning, minor in the second degree and other activities. Research related activities refer to learning activities closely related to research, including doing scientific research with teachers, submitting contributions to professional journals, participating in academic competitions and other activities. Social practice activities refer to learning activities carried out in the off-campus situation that increase students’ social experience or knowledge, including internship, social practice or investigation, community service or volunteer activities. According to the above analysis framework, this study puts forward the following research hypotheses:

H1: undergraduates’ participation in HIPs has a positive and significant impact on learning gains;
H2: different types of HIPs have different effects on different aspects of learning gains.

## Methodology

### Sample technique and procedures

The research sample involved 39 colleges and universities that voluntarily participated in the CCSS project in 2019, including 5 Double First-Class universities, 13 Universities of First-Class Subjects, and 21 local undergraduate colleges. Participating colleges and universities conduct stratified sampling according to grade, gender and subject, and 400–800 students were selected in each grade to participate in the survey. Invalid samples and non-randomly sampled samples were excluded, and cases with missing data in the questionnaire were excluded. The final sample used in this study was 98,218. The sample distribution is shown in Table 1.

**TABLE 1:** Sample distribution.

Name	Category	Number of samples	Proportion (%)	Name	Category	Number of samples	Proportion (%)
Types of institutions	First class university	9933	10.11	Grade	Freshman year	27489	27.99
	First class discipline university	44245	45.05		Sophomore year	26135	26.61
	Other undergraduate institutions	44040	44.84		Junior year	26316	26.79
Subject	Humanities	9930	10.11		Senior year	18278	18.61
	Social discipline	22884	23.30	Gender	Male	49015	49.90
	Science, engineering and medicine	65100	66.28		Female	49187	50.08
	Missing value	304	0.31		Missing value	16	0.02
Student type	A generation of college students	80547	82.01	Student account	Agriculture	47037	48.02
	Non-generation college students	17671	17.99		Non-agricultural	50906	51.98
					Missing value	275	0.28

### Instruments

The data used in this study are from the tracking Survey of China College Student Survey conducted nationwide in 2019 by the Institute of Education of Tsinghua University. Since the national survey was launched in 2009, the CCSS project has been running for more than 10 years. After repeated revision and improvement, the questionnaire has ideal measurement reliability and validity (Tu et al., 2013). It has been widely used in the research fields of college students’ learning engagement, learning satisfaction and analysis of influencing factors of learning gains.

China College Student Survey questionnaire mainly reflects students’ learning experience and educational gains during the school period. The questionnaire is divided into two parts, A and B: Part A mainly reflects the students’ learning experience during the school period, and the item options are 4-point or 7-point Likert scale; Part B is the demographic characteristics of students, family background and other information. According to the research framework, this study selects relevant items in the survey database to construct the required variables.

#### Learning gains

The explanatory variable of this study is the self-reported learning gains of undergraduates. Learning gains refers to the improvement of students’ self-perception in knowledge, ability and values, which is divided into three aspects: knowledge gains, ability gains, and value shaping. The knowledge gains consist of four questions, such as “whether the study and life of the university makes you improve your extensive involvement in various fields of knowledge.” The ability gains consist of eight questions, such as “Has your college life improved your ability to use information technology skillfully.” The shaping of values consists of three questions, such as “Has college life improved your ability to determine your future development plan.” Mplus 18.3 was used for confirmatory factor analysis of learning gains to test its structural validity. It was found that the fitting indexes of learning gains variables were good: *x*^2^ = 40729.93, df = 84, CFI = 0.97 > 0.9, TLI = 0.96 > 0.9, RMSEA = 0.07 < 0.08, SRMR = 0.02 < 0.05. Due to the large sample size, the value of *x*^2^/df was not required. The factor loadings of the learning gain variable measurement items ranged from 0.75 to 0.85. The internal consistency analysis of each dimension showed that the Cronbach’s alpha coefficients were 0.87, 0.94, and 0.86, respectively, exceeding the acceptable level of 0.7, indicating high reliability of the scale.

#### High impact educational practices

The core explanatory variable of this study is HIPs, which include extended learning activities, research related activities and social practice activities. The control variables include students’ individual background variables and institutions’ background variables. Individual background variables include gender, household registration type (agricultural and non-agricultural), and whether they are first-generation college students. First-generation college students refer to students whose parents have not received higher education. College background variables include college support, college type, discipline classification. Among them, college support is a measurement index used by CCSS questionnaire for many years, which measures students’ feelings about the support and help provided by schools in various aspects. It consists of eight questions, such as “providing support and help for students’ studies.” The reliability and validity of this index has been tested by many studies, which is a relatively mature index. In this study, the confirmatory factors of “institutional support” variables were used to test their structural validity. The analysis showed that the model fitting index was good: *x*^2^ = 4197.14, df = 15, CFI = 0.99 > 0.9, TLI = 0.99 > 0.9, RMSEA = 0.05 < 0.08, SRMR = 0.01 < 0.05. The factor loading of each measurement item of the variable was between 0.71 and 0.83, and the Cronbach coefficient was 0.93, which had high reliability. In terms of other control variables, this study divided the types of colleges and universities into three categories: first-class university colleges and universities, first-class discipline colleges and universities, and other undergraduate colleges and universities, while the disciplines were divided into humanities, social sciences, science, engineering, and medicine. In addition, the CCSS questionnaire adopted the method of self-report by students, and the results of self-report were easily affected by the social approval of respondents (Guo et al., 2018). Therefore, social approval was also included in the control variables in the analysis process of this study. Dimensions and descriptive statistics of variables were shown in Table 2.

**TABLE 2:** Constituent dimensions and descriptive statistics of variables.

Variable name and category		Variable definition and measurement method	Mean (standard deviation)/Distribution of categorical variables
**Dependent variable**			
Learning gains	Knowledge gains	The degree of improvement in knowledge perceived by students and the degree of improvement in knowledge perceived by students are composed of four questions.	57.87 (21.65)
	Capacity gains	The improvement degree of students’ perceived knowledge and students’ perceived ability are composed of four questions.	59.65 (21.03)
	Value shaping	The improvement degree of students’ perceived knowledge and students’ perceived values are composed of three questions.	61.78 (22.81)
**Core independent variable**			
HIPs	Extended learning activities	Whether students have participated in any of the three kinds of extended learning activities, dummy variable, 1 = participated, 0 = not participated (control group)	1 = 19.78% 0 = 80.22%
	Research related activities	Whether students have participated in any of the three research related activities, dummy variable, 1 = participated, 0 = not participated (control group)	1 = 31.79% 0 = 68.21%
	Social practice activities	Whether students have participated in any of the three social practice activities, dummy variable, 1 = participated, 0 = not participated (control group)	1 = 65.69% 0 = 34.31%
**Other control variables**			
Types of institutions		The type and category variables of students currently studying, 1 = first-class universities, 2 = first-class discipline universities, 3 = other undergraduate universities.	1 = 10.11% 2 = 45.05% 3 = 44.84%
Institutional support		Students feel that the supportive policies and measures provided by the school for their self-development and success in their studies and employment.	72.66 (17.10)
Subject		The disciplines of students’ current majors are divided into categories and variables, 1 = Humanities, 2 = Social Sciences, 3 = Science, engineering and medicine.	1 = 10.11% 2 = 23.30% 3 = 66.28 Deletion rate = 3.31%
Grade		Students’ current grade, category variable, 1 = freshman, 2 = sophomore, 3 = junior, 4 = senior.	1 = 27.99% 2 = 26.61% 3 = 26.79% 4 = 18.61%
Gender		Dummy variable, 1 = male, 0 = female.	1 = 50.08% 0 = 49.90% Deletion rate = 0.02%
Is it a generation of college students		The students whose parents are educated in high school or below are the first generation of college students. The dummy variable is 1 = the first generation of college students and 0 = the non-first generation of college students	1 = 82.01% 0 = 17.99%
Registered residence		Dummy variable, agricultural household registration = 1, non-agricultural household registration = 0	1 = 47.89% 0 = 51.83% Deletion rate = 0.28%
Social desirability		The situation that individuals are influenced by social expectations and have a high response to self-statement questions consists of 8 questions.	51.80 (21.14)

### Statistical methods

Combined with the research needs, this study first analyzed the situation of students’ participation in HIPs under the background of different types of colleges, gender and disciplines, as well as the differences in learning gains of students’ participation in HIPs. Then, three kinds of HIPs such as expansionary learning activities, research related activities and social practice activities were taken as core explanatory variables. Under the control of gender, grade, household registration and type of institution, regression analysis was used to explore the impact of three kinds of HIPs on learning gains. In this study, Stata13 was used for descriptive statistical analysis and multiple regression analysis. In the analysis process, the sample weight method was used to correct for the difference between the sample and the overall structure.

## Results

### Type differences of undergraduates participating in high impact educational practices

**FIGURE 2:**
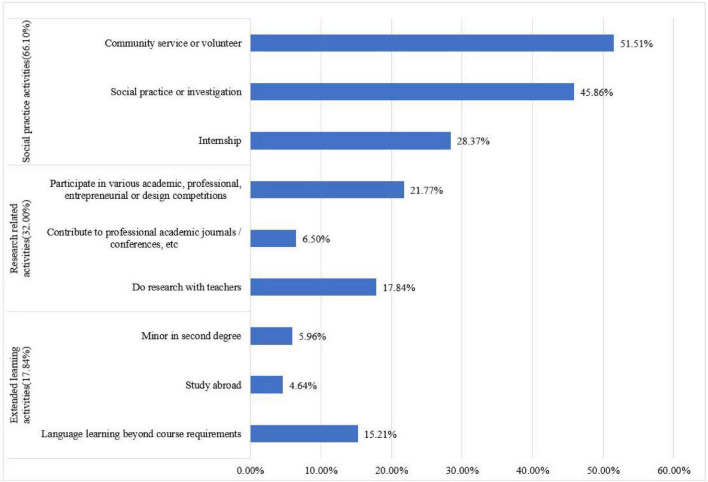
Overall situation of undergraduates participating in high impact education activities.

In general, the proportion of Chinese undergraduates participating in HIPs was not high, and the participation of different types of activities was different. The proportion of participating in any one of extended learning activities, research related activities and social practice activities was 72.11%, and the number of people who had not participated in any HIPs accounted for 27.89%. Among them, the participation rate of social practice activities was the highest (66.10%), followed by research related activities (32.00%), and the participation rate of extended learning activities was the lowest (17.84%). Specifically, **among social practice activities,** community or volunteer activities accounted for the highest proportion (51.51%), followed by social practice or investigation activities (45.86%), and the participation rate of internship activities was the lowest (28.37%). **Among the research related activities,** the proportion of participating in various academic, professional, entrepreneurial, or design competitions was the highest (21.77%), the proportion of doing research with teachers was the second (17.84%), and the proportion of contributing to professional academic journals and conferences was the lowest (6.50%). **Among the extended learning activities,** the proportion of language learning beyond the course requirements was the highest (15.21%), followed by the proportion of minor second degree (5.96%), and the proportion of overseas learning was the lowest (4.64%) as shown in Figure 2.

### Significant background differences of undergraduates’ participation in high impact educational practices

Chi square test was conducted on students’ participation in extended learning activities, research related activities and social practice activities according to institution type, discipline category, grade, gender, student category, and student household registration. The results were shown in Table 3. There were significant background differences of undergraduates’ participation in three types of HIPs. **In terms of the type of institutions,** the proportion of students participating in extended learning activities, research-related activities and social practice activities in first-class universities (25.30, 38.14, and 72.66%, respectively) was higher than that in first-class disciplines universities (20.98, 32.18, and 66.78%, respectively) and other undergraduate universities (17.34% and 29, respectively). **In terms of disciplines,** the proportion of students participating in extended learning activities in humanities (29.53%) was higher than that in social sciences (22.32%) and medical science and engineering (17.35%), and the proportion of students participating in research-related activities in medical science and engineering (32.42%) was higher than that in humanities (31.42%) and social sciences (30.02%). The proportion of students participating in social practice activities in social sciences (69.58%) was higher than that in humanities (66.67%) and science and medicine (64.14%). **In terms of grade,** with the increase of grade, the proportion of female students participating in the three types of HIPs was higher than that of male student. The proportion of non-generation college students and students with non-agricultural household participating in the three types of HIPs was higher than that of the first generation college students and students with agricultural household respectively.

**TABLE 3:** Details of students from different backgrounds participating in HIPs.

Variable	Type	HIPs					
		Extended learning activities	Chi square test	Research related activities	Chi square test	Social practice activities	Chi square test
Types of institutions	First class university	25.30%	396.72***	38.14%	286.59***	72.66%	374.72***
	First class discipline university	20.98%		32.48%		66.78%	
	Other undergraduate institutions	17.34%		29.66%		63.04%	
Discipline category	Humanities	29.53%	930.96***	31.42%	45.52***	66.67%	227.14***
	Social discipline	22.32%		30.02%		69.58%	
	Science, engineering and medicine	17.35%		32.42%		64.14%	
Grade	Freshman year	13.15%	1200***	18.21%	3500***	55.78%	2600***
	Sophomore year	20.33%		33.72%		64.92%	
	Junior year	22.66%		37.73%		67.88%	
	Senior year	24.84%		40.58%		78.58%	
Gender	Male	17.29%	383.27***	30.78%	45.32***	59.47%	1700***
	Female	22.27%		32.97%		71.89%	
Student category	A generation of college students	17.07%	2100***	30.67%	256.65***	65.41%	15.82***
	Non-generation college students	32.17%		36.87%		66.98%	
Student account	Agriculture	14.25%	1700***	29.18%	283.55***	64.70%	41.43***
	Non-agricultural	24.89%		33.49%		66.65%	

***Means at the significant level of 0.001.

### Significant impact of high impact educational practices have a on learning gains and its different dimensions

This study used independent sample *t*-test to analyze the differences in learning outcomes of students’ participation in various HIPs (as shown in Table 4). Data analysis showed that there were significant differences in educational practices between students who participate in extended learning activities, research related activities, social practice activities, and students who did not participate. The average educational practices of students who participated in three types of HIPs were higher than those of students who did not participate. It was found that students’ participation in various HIPs could help improve learning gains.

**TABLE 4:** Analysis of differences in educational practices of students whether they participate in HIPs.

HIPs				
**HIPs**	**Participation**	**Sample size**	**Learning gains**	***t***
			**Mean (standard deviation)**	
Extended learning activities	Participate in	19432	66.81 (20.55)	–55.87***
	Not involved	78783	58.08 (19.23)	
Research related activities	Participate in	31220	67.23 (19.74)	–82.95***
	Not involved	66995	56.35 (18.87)	
Social practice activities	Participate in	64523	62.50(19.89)	–60.06***
	Not involved	33695	54.65 (18.59)	

***Means at the significant level of 0.001.

In order to explore whether different types of HIPs had significant explanatory power and explanatory power on students’ educational practices in different aspects, regression analysis was carried out after controlling students’ individual background and institutional background variables. The regression results were shown in Table 5. It was shown that compared with the baseline models 1, 3, 5, and 7 with only control variables, the explanatory power of models 2, 4, 6, and 8 after adding HIPs was enhanced. In addition, students’ participation in extended learning activities, research related activities and social practice activities had a positive and significant impact on students’ learning gains and different aspects of learning gains.

**TABLE 5:** Regression analysis of HIPs on students’ learning gains.

	Overall gains		Knowledge gains		Capacity gains		Value shaping	
	Model 1	Model 2	Model 3	Model 4	Model 5	Model 6	Model 7	Model 8
Research related activities		0.260***		0.228***		0.268***		0.197***
		(0.012)		(0.012)		(0.011)		(0.011)
Extended learning activities		0.169***		0.152***		0.174***		0.151***
		(0.010)		(0.009)		(0.009)		(0.011)
Social practice activities		0.091***		0.062***		0.095***		0.087***
		(0.009)		(0.011)		(0.010)		(0.008)
Institutional support	0.402***	0.363***	0.362***	0.330***	0.381***	0.340***	0.373***	0.340***
	(0.006)	(0.006)	(0.005)	(0.006)	(0.006)	(0.006)	(0.005)	(0.005)
First class university institutions (dummy variable)	0	0	0	0	0	0	0	0
First class discipline institutions	–0.042*	–0.024	–0.049**	–0.033	–0.049*	–0.030	–0.009	0.006
	(0.024)	(0.031)	(0.018)	(0.023)	(0.026)	(0.032)	(0.026)	(0.032)
Other undergraduate institutions	–0.028	0.010	–0.037*	–0.005	–0.046*	–0.007	0.040	0.073**
	(0.024)	(0.031)	(0.019)	(0.023)	(0.025)	(0.031)	(0.027)	(0.033)
Female student	–0.161***	–0.181***	–0.176***	–0.192***	–0.180***	–0.201***	–0.099***	–0.117***
	(0.009)	(0.007)	(0.008)	(0.007)	(0.011)	(0.009)	(0.008)	(0.008)
A generation of college Students	–0.141***	–0.110***	–0.145***	–0.117***	–0.146***	–0.114***	–0.084***	–0.058***
	(0.009)	(0.007)	(0.011)	(0.009)	(0.009)	(0.007)	(0.008)	(0.007)
Non-agricultural household registration	0.078***	0.056***	0.073***	0.054***	0.095***	0.073***	0.036***	0.018**
	(0.009)	(0.009)	(0.010)	(0.009)	(0.010)	(0.009)	(0.008)	(0.008)
Freshman year (dummy variable)	0	0	0	0	0	0	0	0
Sophomore year	0.123***	0.058***	0.126***	0.070***	0.114***	0.047***	0.073***	0.020*
	(0.011)	(0.011)	(0.013)	(0.012)	(0.011)	(0.011)	(0.010)	(0.010)
Junior year	0.210***	0.127***	0.211***	0.141***	0.185***	0.100***	0.157***	0.091***
	(0.020)	(0.019)	(0.020)	(0.019)	(0.019)	(0.019)	(0.016)	(0.016)
Senior year	0.343***	0.240***	0.363***	0.277***	0.306***	0.200***	0.260***	0.175***
	(0.016)	(0.016)	(0.019)	(0.018)	(0.014)	(0.015)	(0.016)	(0.016)
Humanities (dummy variable)	0	0	0	0	0	0	0	0
Social discipline	–0.018	–0.006	–0.045***	–0.033**	0.024	0.036**	–0.064***	–0.054***
	(0.015)	(0.014)	(0.014)	(0.014)	(0.017)	(0.015)	(0.016)	(0.015)
Science, engineering and medicine	–0.059***	–0.042**	–0.099***	–0.084***	–0.022	–0.004	–0.080***	–0.065***
	(0.017)	(0.016)	(0.017)	(0.017)	(0.019)	(0.017)	(0.016)	(0.015)
Social desirability	0.013***	0.012***	0.012***	0.012***	0.012***	0.012***	0.011***	0.011***
	(0.000)	(0.000)	(0.000)	(0.000)	(0.000)	(0.000)	(0.000)	(0.000)
Sample size	97,621	97,621	97,620	97,620	97,620	97,620	97,620	97,620
*F*	2178	2928	2413	2868	2426	3192	888.8	1226
*R* ^2^	0.306	0.331	0.263	0.282	0.276	0.304	0.242	0.259
Adjusted *R*^2^	0.305	0.331	0.263	0.282	0.276	0.304	0.242	0.259

***Means at the significant level of 0.001, **Means at the significant level of 0.01, *Means at the significant level of 0.05.

Specifically, the impact of research related activities was higher than that of extended learning activities and social practice activities. All three categories of HIPs achieved the greatest improvement on ability gains compared to the other metrics, with a significant increase of 0.268, 0.174, and 0.095 standard deviations (*p* < 0.001), respectively. The impact of participating in research-related activities on knowledge gains (β = 0.228, *p* < 0.001) was higher than that on value shaping (β = 0.197, *p* < 0.001). The impact of participating in extended learning activities on knowledge acquisition and value shaping was similar, which significantly increased by 0.152 and 0.151 standard deviations respectively (*p* < 0.001). The impact of social practice activities on value shaping (β = 0.087, *p* < 0.001) was higher than that on knowledge gains (β = 0.062, *p* < 0.001). The above research results verified the two hypotheses proposed in this study, that was, students’ participation in HIPs had a significant impact on students’ learning gains, and different types of HIPs had different impacts on different aspects of learning gains. The difference value of the impact results should be further analyzed and discussed.

The regression results of models 1, 3, 5, and 7 also showed that institutional support had a significant impact on students’ learning gains (*p* < 0.001), especially on students’ ability gains (β = 0.381, *p* < 0.001) and values shaping (β = 0.373, *p* < 0.001), which showed that the institutional environment played an important role in the development of students. In models 2, 4, 6, and 8 with HIPs, the influence coefficient of institutional support on students’ learning gains decreased, indicating that the influence of institutional support on students’ learning gains might play a role partly by HIPs. In addition, models 2, 4, 6, and 8 showed that compared with first-class universities, other undergraduate universities had significantly higher shaping of students’ values (β = 0.073, *p* < 0.05), but there was no significant difference in students’ learning gains in first-class discipline universities. **In terms of discipline types,** humanities were significantly higher in knowledge gains and value shaping than social sciences and science, engineering, and medicine. Compared with male, female’s learning gains was significantly lower (*p* < 0.001). Compared with non-first-generation college students, first-generation college students were significantly lower in knowledge gains, ability gains and value shaping (*p* < 0.001). Compared with freshmen, senior students had significantly higher educational practices in all aspects (*p* < 0.001).

## Discussion

Based on the analysis of undergraduate students’ learning engagement data, this study investigated the status quo and impact of students’ participation in three types of HIPs.

**Firstly, there are category differences in the participation of Chinese undergraduates in HIPs on the whole.** Among them, the proportion of undergraduates participating in extended learning activities is the lowest, 82.16% of students did not participate in such activities, and 95.36% of students did not participate in overseas learning experience. Participation in social practice activities is slightly better, while 71.63% of students have not participated in internships. The degree of participation in research related activities is not high, and 93.50% of students still have no experience of contributing to professional academic journals/conferences. The above research is basically consistent with a study by Chinese scholars, which found that compared with developed countries such as the United States and South Korea, undergraduates of China generally lacked teacher-student interaction and communication, and their investment in extracurricular learning activities was relatively low (Lv and Zhang, 2015). The reasons for the low participation of Chinese undergraduates in HIPs may be as follows: on the one hand, HIPs are highly challenging and competitive, and students have less opportunities to participate. In particular, overseas study and second-degree study activities are closely related to the school’s ability to supply learning resources. At present, most universities in China are still unable to meet the requirements for students to participate in such activities. On the other hand, the teaching mode of Undergraduate education in China is still in the stage of reform, and the teaching of knowledge as well as skills is still the mainstream. Examination scores are still the main way to evaluate students’ ability. Individualized learning resources for students are insufficient and students are not highly motivated to participate in high-impact educational activities.

**Secondly, there are significant background differences in undergraduates’ participation in HIPs.** From the perspective of institutional background, there are significant differences among different types of universities, grades and disciplines, which shows the dependence of HIPs on institutional conditions. Compared with ordinary colleges and universities, first-class universities and first-class discipline construction colleges and universities can provide more superior resources and conditions. Due to the differences in the training programs of different disciplines and the curriculum settings of different grades, the degree of students participating in HIPs will also vary. From the perspective of family background, female students have a higher degree of participation than male students, which can be attributed to the differences in social development between different genders. The participation of non-first-generation college students is higher than that of first-generation college students, and the participation of non-agricultural college students is also higher than that of agricultural college students, indicating that college students from families with lower economic status are limited by resources and have disadvantages in participating in HIPs, which is consistent with the research of some Chinese researchers (Zhang et al., 2017; Guo, 2019).

**Third, undergraduates’ participation in HIPs helps to improve their learning gains. Different types of HIPs have different impacts on different aspects of students’ gains.** Although there is no clear definition of the concept of HIPs, many studies have verified that HIPs have a positive impact on students’ learning gains to varying degrees. American colleges and universities have also designed and implemented a series of HIPs for undergraduates to improve their basic innovation abilities such as critical thinking, cooperation and communication (Wang et al., 2015). This study found that the three types of HIPs had a positive and significant impact on students’ knowledge gains, ability gains and value shaping. Among them, research related activities had the greatest impact, followed by extended learning activities, and social practice activities had less impact. However, from the current situation, the proportion of students participating in the first two types of activities is low. Previous studies have shown that institutional support had a direct positive impact on students’ learning gains, that was, the more resources the school provided, the better the supportive policy guarantee, the more students would gain (He, 2016). In view of the high demands on physical and human resources for research-related activities and extended learning activities, the promotion of students’ participation in these two types of activities requires the creation of a more supportive environment and the provision of more resources and opportunities for participation. From different aspects of educational practices, the three types of HIPs had the greatest impact on students’ ability gains. This showed that for undergraduates at this stage, participating in HIPs had become an important learning method to improve their comprehensive ability. HIPs emphasize learning autonomy, cooperation and inquiry, which can stimulate students’ learning in the way of in-depth learning, thus profoundly affecting the overall development of students.

## Limitations

The limitations of this study are related to the samples and methods used in the study. Undergraduates from 39 colleges and universities in China were selected for the study, but different colleges and universities have different resources and abilities to provide high impact educational activities. There will be differences in the understanding of high impact educational activities. This situational difference will affect the research results to a certain extent. In terms of the definition and classification of high impact educational activities, the research lacks qualitative research design, and interviews with different groups of people need to be increased. In addition, the classification of high impact educational activities in the research is carried out on the basis of the questionnaire items, which may cause problems that the classification and definition of high impact educational activities are not necessarily appropriate. In the follow-up research, it is necessary to refine and clarify the connotation of high impact educational activities in combination with the practical situation of high impact educational activities in Chinese universities.

## Implications and future work

This study shows the basic situation of Chinese undergraduates’ participation in high-impact educational activities, and confirms the importance of high-impact educational activities for student development through data. Research shows that it is quite necessary to promote students’ participation in high-impact educational activities. Students gain knowledge through participating in high-impact educational activities, improve their personal comprehensive ability, and also affect the formation of values. How to promote students’ participation in high-impact educational activities and how to provide more participation in high-impact educational activities are issues that need to be considered in the development of universities. The biggest challenge in the research is how to understand the concept of high-impact educational activities. In future research, we should grasp the essential characteristics of high-impact educational activities and avoid the risk of conceptual generalization. At the same time, considering the classification and definition of high-impact educational activities in different cultural backgrounds, more field interview materials should be added in future research to explore the understanding of different groups of people in cultural backgrounds on high impact educational activities, so as to define them more clearly.

This study mainly explores the role of high-impact educational activities from the perspective of external behavior, and further research is needed on the motivation of students’ internal participation in activities and its influencing mechanism. Whether students participate in influential educational activities or what type of high-impact educational activities they can participate in will be affected by factors such as students’ individual learning motivation, interests, family economic and cultural capital, and college environment and resources, and these factors will also affect students’ learning gains. In future research, more potential influencing factors should be included for analysis, and more samples should be adopted to analyze the degree of influence of high-impact educational activities based on students’ learning motivation, family background, college environment and other factors.

In the future research, it is planned to use the hybrid research method of exploratory timing design. First, the grounded theory is used to summarize and refine the concept of high impact educational activities in combination with the learning situation and cultural situation of Chinese college students, and the high impact educational activities are classified more accurately, and then analyzed through large-scale questionnaire survey data. In the future, the most important thing is how to change the teaching concept of universities, increase the resources of high impact educational activities and provide sufficient support, so as to give full play to the educational function of high impact educational activities.

## Conclusion

This paper explores the impact of undergraduate students’ participation in high-impact educational activities on learning outcomes, as well as the impact of different types of high-impact educational activities on different aspects of learning outcomes. The study concluded that students’ participation in high-impact educational activities has a positive impact on learning outcomes, and three types of high-impact educational activities, including extended learning activities, research-related activities, and social practice activities, have positive effects on knowledge acquisition, ability improvement, and social practice. The impact on the shaping of values is different. At present, most studies on high-impact educational activities focus on the first generation of college students in their families, and most of them are conducted on the population and samples of western countries. Under the background of different educational and cultural environments, the understanding of high-impact educational activities may exist. This background triggered the implementation of this study to further develop the impact of different types of high-impact educational activities on student development in different cultural contexts, thereby increasing the universality of the classification of high-impact educational activities.

## Data availability statement

The raw data supporting the conclusions of this article will be made available by the authors, without undue reservation.

## Author contributions

HK designed the study. HK, NJ, LX, and ZX analyzed the data and wrote and modified the manuscript. All authors have read and agreed to the published version of the manuscript.
